# Bone scan index (BSI) scoring by using bone scintigraphy and circulating tumor cells (CTCs): predictive factors for enzalutamide effectiveness in patients with castration-resistant prostate cancer and bone metastases

**DOI:** 10.1038/s41598-023-35790-5

**Published:** 2023-05-29

**Authors:** Hisashi Hirano, Masayoshi Nagata, Naoya Nagaya, So Nakamura, Takeshi Ashizawa, Yan Lu, Haruna Kawano, Kosuke Kitamura, Yoshiro Sakamoto, Kazuhiko Fujita, Hideyuki Isobe, Akira Tsujimura, Satoru Muto, Shigeo Horie

**Affiliations:** 1 Department of Urology, Juntendo University Graduate School of Medicine, 2-1-1 Hongo Bunkyo-Ku, Tokyo, 1138431 Japan; 2 Department of Urology, Juntendo University Nerima Hospital, Tokyo, Japan; 3 Department of Urology, Juntendo University Shizuoka Hospital, Shizuoka, Japan; 4 Department of Urology, Juntendo Tokyo Koto Geriatric Medical Center, Tokyo, Japan; 5 Department of Urology, Juntendo University Urayasu Hospital, Chiba, Japan

**Keywords:** Prostate cancer, Cancer therapeutic resistance, Bone metastases

## Abstract

Reports of Bone Scan Index (BSI) calculations as imaging biomarkers to predict survival in patients with metastatic castration-resistant prostate cancer (mCRPC) have been mainly from retrospective studies. To evaluate the effectiveness of enzalutamide (ENZ) in Japanese patients with mCRPC and bone metastases using BSI (bone scintigraphy) and circulating tumor cell (CTC) analysis. Prospective, single-arm study at Juntendo University affiliated hospitals, Japan. Patients were administered 160 mg ENZ daily, with 3 monthly assessments: BSI, prostate specific antigen (PSA), CTC and androgen receptor splicing variant-7 (AR-V7) status. Primary endpoint: BSI-decreasing rate after ENZ treatment. Secondary endpoints: PSA-decreasing rate and progression free survival (PFS). Statistical analyses included the Wilcoxon t-test, Cox proportional hazard regression analysis, and log-rank test. Median observation period: 17.9 months, and median PFS: 13.8 (2.0–43.9) months (n = 90 patients). A decrease in BSI compared to baseline as best BSI change on ENZ treatment was evident in 69% patients at the end of the observation period (29% patients showed a complete response, BSI 0.00). At 3 months 67% patients showed a ≥ 50% PSA reduction, and 70% after ENZ treatment. PSA decline (3 months) significantly associated with a prolonged median PFS: 18.0 (estimated) versus 6.4 months (HR 2.977 [95% CI 1.53–5.78], *p* = 0.001). Best BSI decline response significantly associated with a prolonged PFS: 18.1(estimated) versus 7.8 months (HR 2.045 [95% CI: 1.07–3.90], *p* = 0.029). CTC negative status (n = 20) significantly associated with a prolonged PFS: 13.4 [estimated] vs 8.6 months (HR 2.366, 95% CI 0.97–5.71, *p* = 0.041). CTC positive/AR-V7 positive status significantly associated with a shorter PFS: 5.9 months (HR 8.56, 95% CI 2.40–30.43, *p* = 0.0087). -reduction (3 months) and BSI-reduction (on ENZ treatment) were significant response biomarkers, and a negative CTC status was a predictive factor for ENZ efficacy in patients with mCRPC.

## Introduction

Enzalutamide (ENZ) targets multiple steps in the androgen receptor signaling pathway responsible for prostate cancer growth^1^. Enzalutamide prolongs progression free survival (PFS) and overall survival (OS) in patients with metastatic castration-resistant prostate cancer (mCRPC) before and after chemotherapy^2,3^, and has been available in Japan for the treatment of mCRPC since 2014.

The majority of patients with CRPC eventually present with bone metastases, and evaluating bone metastases is an important factor in determining prognosis for these patients^4^. Bone metastases in patients with mCRPC are generally osteoblastic or mixed-type. While bone scintigraphy imaging may be poor at delineating osteolytic lesions*,* it is useful for osteoblastic lesions. This type of imaging used with bone scan index (BSI) calculations is an objective parameter for evaluating bone metastases, and therefore has usefulness as a prognostic marker in patients with mCRPC^5,6^. Areas where bone accumulation is high in scintigraphy can be detected and counted using BSI. Bone scan index calculations are quantitatively expressed as a ratio of metastatic lesions in the entire bone area^7^. While there are some reports of the usefulness of BSI in clinical practice as an imaging biomarker to predict survival for patients with mCRPC, these have been mainly from retrospective studies^8–12^.

Another potential prognostic marker to monitor treatment effectiveness in patients with mCRPC is the use of non-invasive liquid biopsies. These are blood-based, non-solid biological tissues such as circulating tumor cells (CTCs), exosomes, and cell-free DNA. Circulating tumor cells can be characterized as cancer cells that intravasate the circulatory system from primary locations^13^. In patients with mCRPC, the presence of androgen receptor splice variant 7 (AR-V7) in CTCs is a potential biomarker to predict the development of drug resistance to ENZ and abiraterone^14,15^. Unlike antiandrogen treatments, taxane-based chemotherapies are effective in mCRPC irrespective of AR-V7 status^16,17^. Data from our previous study have also shown these findings in the Asian population^18^. Therefore, to optimize treatment selection and avoid debilitating side effects, genetic analysis of CTCs has the potential to individualize patient treatment in mCRPC.

In this prospective, single-arm study, we evaluated the effectiveness of ENZ in Japanese patients with mCRPC and bone metastases using BSI from bone scintigraphy and CTC analysis.

## Materials and methods

### Patients and study design

A total of 100 patients with mCRPC were treated at the Juntendo University affiliated hospitals in Japan between July 2015 to December 2020. The patient registration period was from 2015 to 2018, with an observation period up to the end of 2020. All registered patients presented with bone metastases and were administered 160 mg ENZ daily.

Blood sampling and bone scintigraphy was conducted every 3 months. Collection of CTCs was performed before ENZ administration and every 3 months for the 46 patients with mCRPC at Juntendo University Hospital.

Baseline parameters included: age and Gleason score (GS) at the time of diagnosis of prostate cancer, time to CRPC, metastatic lesions, laboratory findings, previous treatments, and use of bone modifying agents (BMAs).

Docetaxel pretreatment was recorded; however, in Japan upfront docetaxel was generally not used for metastatic hormone-sensitive prostate cancer during the study period.

The treatment policy for the use of BMAs at Juntendo University affiliated hospitals was followed. In principle, all patients irrespective of whether prostate cancer was hormone-sensitive or castration-resistant received BMAs, such as denosumab and zoledronic acid, at the time they were diagnosed with bone metastases. However, if osteonecrosis of the jaw or dental caries requiring invasive treatment developed after use, BMAs were promptly discontinued. The decision to use either denosumab or zoledronic acid was made at the discretion of the attending physician.

### Study endpoints

The primary endpoint was BSI-decreasing rate after ENZ treatment. Secondary endpoints included: prostate specific antigen (PSA) response rate, and PFS. Landmark PFS analyzes at 6 months and 18 months on ENZ treatment were conducted for change in BSI or PSA. Other parameters evaluated included detection of CTCs, and AR-V7 status from CTCs.

The effectiveness of ENZ on bone metastases, measured by BSI, was assessed as best BSI response during the overall treatment period. The overall treatment period was chosen as treatment flare-up is possible after 3 months of ENZ treatment.

Progressive Disease (PD) was assessed as disease exacerbation on bone scintigraphy and computerized tomography (CT), as well as PSA elevation (> 25% and > 2 ng/mL above nadir); these patients were offered other treatment options. The PFS was defined as time from start of ENZ treatment to the failure to continue ENZ due to PD.

### CTC analyses

CTC analysis using peripheral blood was performed as a subgroup analysis. However, CTC analysis could only be carried out at the main center (Juntendo University Hospital) as prompt analysis is required after blood collection.

These methods have been previously reported^14,15,18,19^. In brief, the AdnaTest (QIAGEN, Germany) was used to detect CTCs in accordance with the manufacturer’s protocol. Blood (5 ml) was collected into EDTA-3K and RNA extracted using antibody-conjugated magnetic beads (AdnaTest ProstateCancerSelect). Extracted mRNA (AdnaTest ProstateCancerDetect) was then subjected to reverse transcription using the Sensiscript Reverse Transcriptase Kit (QIAGEN). Reverse transcription polymerase chain reaction (RT-PCR) was used to detect expression of PSA, androgen receptor (AR), and AR-V7 in CTCs. The AdnaTest PrimerMix ProstateDetect was used for amplification of PSA (PCR conditions for PSA: 95 °C for 15 min, 42 cycles at 94 °C for 30 s, 61 °C for 30 s, 72 °C for 30 s, followed by 10 min of extension). When samples were positive for PSA, we defined successful CTC detection as positive PSA expression. The AdnaTest PrimerMix AR-Detect was used for amplification of AR (PCR conditions for AR: 95℃ for 15 min, 35 cycles at 94 °C for 30 s, 60 °C for 30 s, 72 °C for 60 s, followed by 10 min of extension). The primer set and PCR conditions for AR-V7 RT-PCR were as follows: the AR-V7 primer set was designed to yield 125-bp AR-V7-specific band, 5′-CCATCTTGTCGTCTTCGGAAATGTTA-3′, and 5′-TTTGAATGAGGCAAGTCAGCCTTTCT-3′ (PCR conditions for AR-V7: 95 °C for 5 min, 39 cycles at 95 °C for 10 s, 58 °C for 30 s, 72 °C for 30 s, followed by 10 min of extension). The amplified PCR products were electrophoresed and visualized using the DNA 1K Experion automated electrophoresis system (Bio-Rad, CA, USA). To evaluate gene expression, the fluorescence intensity scale was set to “scale to local” (default setting), and any visible bands with detectable peaks under these conditions were considered positive for AR-V7.

### Statistical analyses

Statistical analyses were performed using the Fisher’s exact test for categorical variables, the Wilcoxon t-test, and Mann–Whitney U-test for continuous variables. Kaplan–Meier plots were used for PFS analyses, and differences compared with the log-rank test. Univariate and multivariate analyses were performed using multiple regression analysis and the Cox proportional hazard model.

Patients who were CTC positive before ENZ administration were compared with the CTC negative group, and PFS correlation examined using the log-rank test.

Statistical significance was defined as *p* < 0.05. All statistical analyses were performed with the EZR software for medical statistics, which is based on R and a modified version of R commander designed to add statistical function, and frequently used in biostatics^20^.

### Ethics

This study was approved by the institutional review board of Juntendo hospital (Admission Number: 15-060, 14-052), and all experiments were carried out in accordance with approved guidelines. All participants submitted written informed consent. This study was also registered in the UMIN (University Hospital Medical Information Network) in Japan (UMIN ID: UMIN000018634).

## Results

Out of the 100 patients registered in this study, 10 patients withdrew: patient request for withdrawal (n = 2), discontinuation due to other diseases (acute myocardial infarction, n = 2; cerebral hemorrhage, n = 1), and lost-to-follow-up (n = 5). A total of 90 patients with mCRPC were treated with ENZ; there were no ENZ treatment discontinuations due to adverse events. The median observation period was 17.9 months.

Patient characteristics at the time of prostate cancer diagnosis are shown in Table 1a. The median age at diagnosis was 74.0 ± 7.6 years, and the median initial PSA value was 1300 ± 2786.1 ng/mL. The Gleason score at diagnosis was ≥ 8 in 78% (70/90 patients). At first diagnosis, 84% (76/90 patients) had de novo metastatic prostate cancer with bone metastases (bone metastases developed in the remainder of patients during treatment). In 82% (74/90 patients), initial treatment was hormone therapy including combined androgen blockade (CAB). Although in 12 patients, local treatments, such as surgery or radiotherapy were performed as initial treatments.

**Table 1:** Patient characteristics. (a) Characteristics at diagnosis of prostate cancer. (b) Characteristics before enzalutamide administration.

N = 90	
(a) Characteristics at diagnosis of prostate cancer	
Median age (± SD)	74.0 ± 7.6
Median BMI (± SD)	23.0 ± 3.5
Median initial PSA (± SD)	1300 ± 2786.1
Gleason score	
3 + 3	2
3 + 4	2
4 + 3	16
4 + 4	40
4 + 5	24
5 + 4	4
5 + 5	2
Bone metastases at diagnosis	76
Initial therapy	
CAB	74
Surgical castration	4
Radical prostatectomy	7
Radiation	3
CAB + radiation	2
(b) Characteristics before enzalutamide administration	
Median time-to-CRPC (month) (± SD)	34.4 ± 35.7
Median PSA (± SD)	72.3 ± 183.5
Bone modifying agents (BMAs)	
Denosumab	66
Zoledronic acid	4
Therapies after CRPC diagnosis	
No previous therapy (ENZ first-line)	44
AAT	33
Abiraterone	14
Docetaxel	21
Radium-223	2
Metastases	
Lymph nodes	25
Lung	13
Liver	3

*AAT* alternative nonsteroidal anti-androgen therapy, *BMI* body mass index, *CAB* combined androgen blockade, *CRPC* castration-resistant prostate cancer, *PSA* prostate-specific antigen, *SD* standard deviation.

Table 1b shows patient characteristics before ENZ administration. The median (range) time-to-CRPC was 34.4 (1.0–146.5) months, and median (SD) PSA value before ENZ administration was 72.3 ± 183.5 ng/m. Visceral metastases, other than bone metastases, were observed in 17% (15/90 patients; one patient had both liver and lung metastasis) before ENZ administration. In 49% (44/90 patients), ENZ was used as a first-line treatment for mCRPC, and BMAs were used in 78% (70/90 patients).

**Figure 1:**
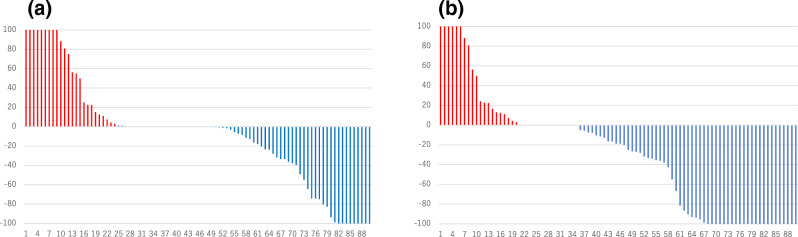
Waterfall plots of BSI change rate and best BSI change while on ENZ treatment at the end of the observation period. (**a**) BSI change rate at 3 months after ENZ administration. (**b**) Best BSI change while on ENZ treatment at the end of the observation period. As a primary endpoint, 69% (62/90 patients) showed a decrease in BSI at the end of the observation period compared to baseline as best BSI change while on ENZ treatment, and 47% (42/90) of patients achieved a BSI reduction after 3 months of ENZ administration. 20% of patients (18/90) showed a ≥ 50% reduction in BSI using bone scintigraphy at 3 months after ENZ administration (10% of patients achieved a complete response), and 38% of patients (33/90) showed a ≥ 50% best BSI response during the course of ENZ treatment (29% of patients achieved a complete response). BSI, bone scan index; ENZ, enzalutamide.

The median (range) PFS was 13.8 (2.0–43.9) months (n = 90). For the primary endpoint (BSI-decreasing rate), 69% (62/90 patients) showed a decrease in BSI at the end of the observation period compared to baseline, as the best BSI change while on ENZ treatment. A total of 42/90 patients (47%) achieved a BSI reduction after 3 months of ENZ administration. At 3 months after ENZ administration, 20% (18/90 patients) showed a ≥ 50% reduction in BSI, and 10% (9/90 patients) achieved a complete response (CR) i.e. BSI value 0.00 on bone scintigraphy. A total of 38% (33/90 patients) demonstrated a ≥ 50% reduction at the end of the observation period compared to baseline as the best BSI change on treatment, with 29% (26/90 patients) achieving a CR (Fig. 1). Clinical factors that made a significant contribution to a reduction in BSI included low alkaline phosphatase (ALP) levels after 3 months of ENZ treatment (*p* = 0.004), and concomitant use of BMAs, such as zoledronic acid or denosumab (*p* = 0.041; Table 2).

**Table 2:** Univariate analyses of clinical factors in patients with, or without BSI reduction while on treatment at the end of the observation period after enzalutamide administration.

	*P* value
Age	0.737
BMI	0.543
Best PSA response after ENZ	0.281
Initial PSA	0.19
ALP at 3 months after ENZ	**0.004**
Time-to-CRPC	0.753
Concomitant use of BMAs	**0.041**
Local therapy before ENZ	0.182
AAT before ENZ	0.777
Docetaxel before ENZ	0.351
Abiraterone before ENZ	0.233
Liver metastases	1.01
Lung metastases	1.00
Lymph nodes metastases	0.565

Two clinical factors significantly correlated with a decrease in BSI compared to baseline as best BSI change while on ENZ treatment at the end of the observation period: low ALP levels at 3 months after ENZ treatment (*p* = 0.004), and concomitant use of BMAs (*p* = 0.041).

*AAT* alternative nonsteroidal anti-androgen therapy, *ALP* alkaline phosphatase, *BMA* bone modifying agents, *BMI* body mass index, *BSI* bone scan index, *CRPC* castration-resistant prostate cancer, *ENZ* enzalutamide, *PSA* prostate-specific antigen.

Statistically significant differences are shown in bold.

Treatment with ENZ was effective and continued in 48 patients (53%) during the observation period. A total of 42 patients (47%) became ENZ-refractory and discontinued treatment during the observation period. There were no significant differences in patient characteristics between the ENZ continuation and ENZ discontinuation groups (Supplementary Table 1). However, univariate analyses revealed a significant difference between these two groups for PSA decline (< 32 ng/mL) at 3 months after ENZ treatment (*p* = 0.019), and a decrease in BSI at the end of the observation period compared to baseline as best BSI change on therapy (*p* = 0.031), Supplementary Table 2. The median PSA value at 3 months for all 90 patients was 32 ng/mL, and this was set as the cut-off value.

**Figure 2:**
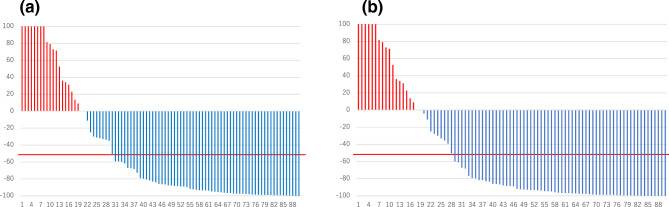
Waterfall plots of PSA change rate and best PSA change while on treatment at the end of the observation period. (**a**) PSA change rate at 3 months after ENZ administration. (**b**) Best PSA change while on treatment at the end of the observation period after ENZ administration. 67% of patients (60/90) showed a ≥ 50% reduction in PSA at 3 months after ENZ administration compared to baseline, and 70% of patients (63/90) showed a ≥ 50% reduction as best PSA change while on treatment at the end of the observation period after ENZ treatment. The red line indicates a − 50% reduction. ENZ, enzalutamide; PSA, prostate-specific antigen.

At 3 months after ENZ administration, 67% (60/90 patients) showed a ≥ 50% reduction in PSA compared to baseline, and 70% (63/90 patients), showed a ≥ 50% reduction at the end of the observation period as best PSA change while on ENZ treatment (Fig. 2).

Three further subgroup analyses were conducted, including: BSI and PSA reduction rates in patients with visceral metastases, patients treated with docetaxel chemotherapy before ENZ treatment, and in two further patient groups, with and without BMA use at baseline. BSI reduction was defined as a decrease in BSI at the end of the observation period compared to baseline as best BSI change while on ENZ treatment. The PSA-reduction rate was evaluated 3 months after ENZ administration. There were no notable differences in patient background and clinical characteristics for patients with and without visceral metastasis (Supplementary Table 3). A reduction in BSI was observed in 80% (12/15 patients) with visceral metastases (Supplementary Fig. 1). There was a decrease in PSA of 87% (13/15 patients). Furthermore, BSI reduction was observed in 62% (13/21 patients) treated with docetaxel chemotherapy before ENZ treatment, and PSA decreased by 71% (15/21) in these patients (Supplementary Fig. 2). The mean follow-up period for ENZ treatment in the post-docetaxel group was 14.5 months, and the median PFS for this group was 9.5 (2.5–20.0) months. A reduction in BSI was observed in 69% (48/70 patients) with BMA use, and 60% (12/20 patients) without BMA use at baseline (Supplementary Fig. 3).

**Figure 3:**
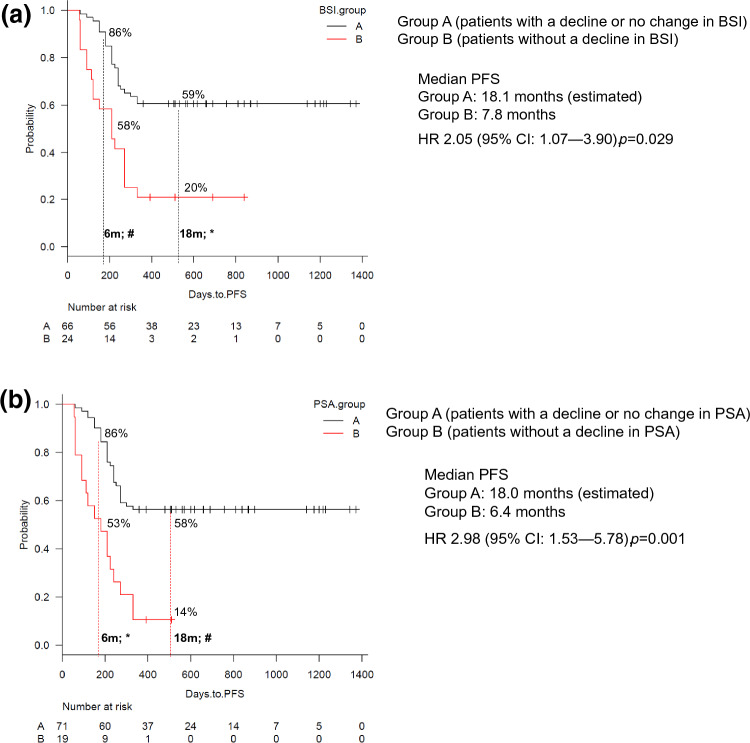
Kaplan–Meier Curve of progression free survival (PFS) rate in patients with mCRPC and bone metastases after enzalutamide administration. (**3**) PFS after enzalutamide administration in Group A (patients with a decline or no change in BSI) and Group B (patients without a decline in BSI), as best BSI change while on treatment compared with baseline. (**b**) PFS at 3 months after enzalutamide administration in Group A (patients with a decline or no change in PSA) and Group B (patients without decline in PSA). As best BSI change while on treatment at the end of the observation period, 73% (66/90 patients) showed a decrease or no change in BSI compared to baseline after ENZ treatment. At 3 months after ENZ treatment, 79% (71/90 patients) showed a decrease or no change in PSA. The median observation period was 17.9 months for all 90 patients. Dotted lines indicate landmark points at 6 and 18 months, percentage of progression-free cases. BSI, bone scan index; CRPC, castration-resistant prostate cancer; ENZ, enzalutamide; PSA, prostate-specific antigen; *p < 0.05; #not significant difference.

For best BSI change on therapy at the end of the observation period, 73% (66/90 patients) showed a decrease, or no change in BSI compared to baseline after ENZ treatment. At the end of the observation period, this group also demonstrated significant association with a prolonged PFS: median 18.1(estimated) months vs 7.8 months (HR 2.05 [95% CI 1.07–3.90], *p* = 0.029; Fig. 3a). At 3 months after ENZ treatment, 79% (71/90 patients) showed a decrease or no change in PSA. Any decline or no change in PSA at 3 months after ENZ treatment was significantly correlated with prolonged PFS: median 18.0 (estimated) months versus 6.4 months (HR 2.98 [95% CI 1.53–5.78], *p* = 0.001; Fig. 3b). Landmark PFS analyses at 6 and 18 months were 86% and 59%, respectively (BSI-reduction group), and 58% and 20%, respectively in the non BSI-reduction group. Further PFS rates were 86% (6 months) and 58% (18 months) for the PSA declining group compared to 53% and 14% in the non-PSA declining group. The difference in PFS between these two groups was statistically significant at 18 months (with and without BSI decline; *p* = 0.045), and at 6 months (with and without PSA decline; *p* = 0.029), (Fig. 3a,b).

**Figure 4:**
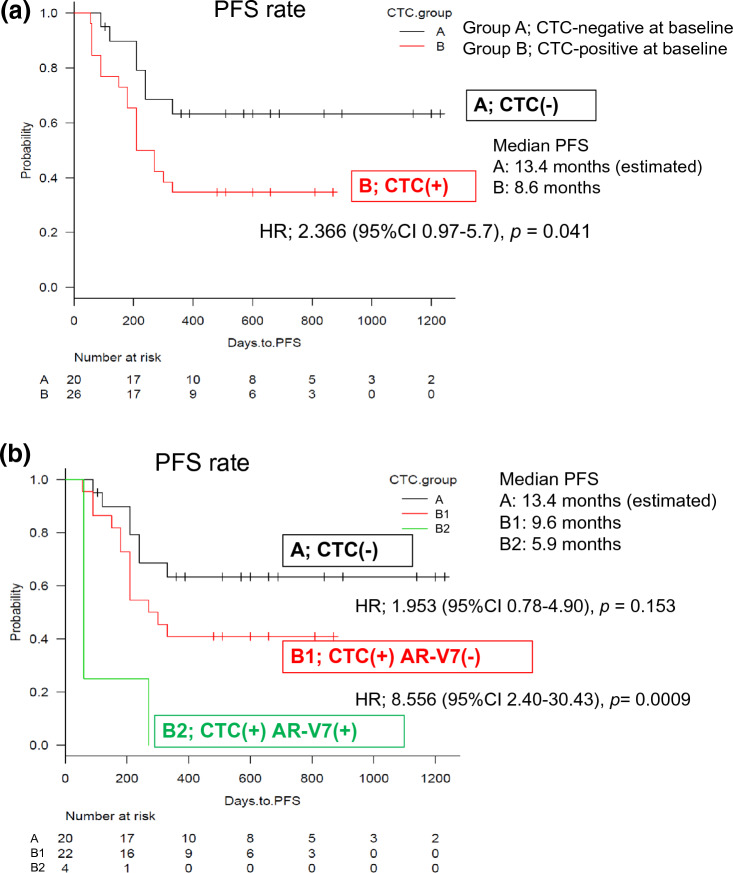
Kaplan–Meier Curve of progression-free survival rate (PFS) of mCRPC patients with bone metastases. (**a**) Correlation of PFS with and without CTC at baseline. Group A; the CTC-negative, Group B; the CTC-positive before ENZ-administration. The PFS were analyzed statistically log-rank test. (**b**) Correlation of PFS with and without AR-V7 in CTC at baseline. Group A; CTC-negative before ENZ-treatment, Group B1; CTC-positive and AR-V7-negative, Group B2; CTC-positive and AR-V7-positive. The PFS were analyzed statistically log-rank test.

Blood samples from the 46 patients treated at Juntendo University Hospital were analyzed for CTCs prior to ENZ administration. Due to the time-sensitive nature of CTC analysis, the remaining 44 samples collected at the affiliated hospitals were not analyzed. There were no differences in patient background and clinical characteristics for patients at Juntendo University Hospital and the affiliated hospitals (Supplementary Table 4). A total of 26 patients from Juntendo University Hospital were CTC positive, and 20 patients were CTC negative. Progression free survival was significantly longer in the CTC negative group (median 13.4 [estimated] months) vs the CTC positive group (median 8.6 months); HR 2.37, 95% CI 0.97–5.71, *p* = 0.041), as shown in Fig. 4a. Of the 26 patients who were CTC positive, 22 patients were AR-V7 negative and 4 patients were AR-V7 positive. Progression free survival was significantly shorter in patients who were CTC positive/AR-V7 positive (median 5.9 months, HR 8.56, 95% CI 2.40–30.43, *p* = 0.0009) (Fig. 4b).

Overall, ENZ was well tolerated; there were no serious adverse events, or adverse events leading to treatment discontinuation during the observation period. The dose of ENZ was reduced in 4 patients due to poor appetite and malaise. There were no treatment-related deaths.

## Discussion

This prospective study demonstrated the efficacy of ENZ in 90 Japanese patients with mCRPC and bone metastases. The reduction in BSI during ENZ treatment was significant response biomarkers for ENZ efficacy in patients with mCRPC. While a decline in PSA at 3 months after ENZ treatment was also significantly correlated with prolonged PFS, a reduction in BSI compared to baseline, as best BSI change while on treatment occurred in 62% of patients (29% CR) at the end of the observation period versus 42% of patients at 3 months. One of the reasons for a later BSI decline could be due to the “flare-up” phenomenon. Studies in patients with metastatic prostate cancer have shown that flares peak on bone scintigraphy 6 to 8 weeks after treatment^22,23^, and as a result of this, the Prostate Cancer Working Group 2 (PCWG2) do not recommend any immediate changes to treatment if there is an increase in bone accumulation using scintigraphy, or an increase in PSA levels within 12 weeks of treatment initiation^24^. While 7 patients in this study presented with a slight, temporary exacerbation of BSI at 3 months after ENZ administration, all patients improved at 6 months, and this was considered to be a flare-up effect. If BSI is evaluated within 3 months of ENZ administration, caution is required when interpreting results to determine whether disease exacerbation has actually occurred. Thereby, to exclude any influence of the flare-up effect and accurately evaluate ENZ effectiveness on bone metastases using bone scintigraphy, BSI evaluation after 6 months is preferable.

Best PSA response rate after ENZ treatment (≥ 50% reduction in PSA) in 70% of patients was comparable to 54% of patients in the AFFIRM study (ENZ post-docetaxel) and 78% in the PREVAIL study (ENZ pre-docetaxel)^2,3^. Median PFS (13.8 months) was also comparable to AFFIRM (8.3 months) and PREVAIL (14 months)^2,3^. Early PSA decline at 3 months (67% of patients) was significant for predicting ENZ treatment effectiveness; this was also reported in the PREVAIL trial where PSA decline within 3 months was associated with a greater likelihood of 5-year survival^21^.

Two clinical factors showed a significant contribution to BSI reduction: ALP levels at 3 months after ENZ treatment, and the concomitant use of BMAs. In this study, a decrease in ALP was predictive of ENZ effectiveness on bone metastases prior to diagnostic imaging. Although the efficacy of BMAs in the prevention of bone-related events has been well established in patients with metastatic prostate cancer, its survival benefits are not generally known^25–27^. Findings from this study suggest that BMAs might contribute to the effectiveness of ENZ treatment on bone metastases. However, if BSI reduction rate data are considered for patients with and without BMA use at baseline (Supplementary Fig. 3), together with the aim of BMA therapy in preventing bone-related events, it is difficult to evaluate whether BMAs are a beneficial add-on to ENZ efficacy for bone metastases.

The line of treatment for ENZ was not consistent during this study, with ENZ was used as first-line therapy in 44% (44/90 patients). As a result, previous treatments may have contributed to the efficacy of ENZ. Taking this into consideration, a subgroup analysis was performed in patients who had undergone docetaxel chemotherapy prior to ENZ treatment. However, these findings suggest that the response of ENZ treatment was not very low even for cases after docetaxel although the number of cases was small in this study (Supplementary Fig. 2).

While CTC analysis was just performed on a small number of patients, a reduction in PFS was clearly evident in patients who were CTC positive. Of the 24 patients who had samples analyzed by liquid biopsy at baseline and became ENZ-refractory, 71% (17/24 patients) were CTC positive. The detection of CTCs, in particular AR-V7, can help to predict treatment resistance in patients with prostate cancer^14,15,28^. The decrease or disappearance of CTCs 3 months after treatment in patients with mCRPC has been shown to be a biomarker for predicting a stronger therapeutic effect than a decrease in PSA^29^. According to recent findings, the number of CTCs should be considered when describing the relationship between AR-V7 status and prognosis in CTCs^30^. Even though the AdnaTest method was negative for CTCs, the CellSearch method detected 69.5% CTCs^30^; this suggests that the AdnaTest method is less sensitive than the CellSearch method. However, in this study, we used the AdnaTest as a Point of Care Testing (POCT) method, focusing on a simple and feasible way of carrying out a CTC analysis. We demonstrated that AR-V7 in CTCs can be detected in any laboratory without the need for expensive equipment, and that results can be applied in the context of precision medicine. The AdnaTest may also be useful in clinical practice as a POCT tool to evaluate AR-V7 status even if sensitivity for CTC detection is slightly inferior compared to the CellSearch method.

Enzalutamide was generally well tolerated, and there were no treatment discontinuations due to AEs. An AE frequency (≥ Grade 3) of 23% has been reported with ENZ treatment^3^; and it has been suggested that Japanese patients may be more tolerant towards ENZ; however, this remains to be elucidated.

This study has several limitations. Firstly, due to the short observation period, the number of fatal events was small, and OS was not evaluated. This was a single-arm study with no control group. Secondly, for patients with a small amount of bone metastases (BSI ≤ 1%), false positive effects, such as an accumulation in jaw osteonecrosis lesions and benign bone degeneration, were possible. Thirdly, visceral metastases, including liver and lungs were present in 18% of patients, and to truly evaluate the effectiveness of ENZ on bone metastases, it would be desirable to exclude patients with visceral metastases. However, a subgroup analysis in the 15 patients with visceral metastases (Supplementary Table 3 and Supplementary Fig. 1), suggest the possibility that ENZ may have equivalent effectiveness in this population. Fourthly and most importantly, the AdnaTest was used exclusively for CTC collection and analyses. If a highly sensitive method was used to count CTCs, such as CellSearch, the number of CTC-negative groups would be considerably less. The Adna Test method may have also contributed to the higher survival rate in the CTC-negative group. Finally, CTC analyses could only be performed for patients at Juntendo University Hospital due to time-sensitive sample processing. As a result CTC analysis was only performed on half of all patients; this combined with the number of events, and analysis period may have been insufficient for an accurate evaluation.

It is important to note, however, that compared to tissue biopsy, which is a relatively invasive procedure, CTC analysis is far less invasive and can be performed at regular time intervals. Monitoring CTCs during treatment may be a more practical option in the clinic. In a liquid biopsy, circulating tumor DNA (ctDNA), circulating exosomes as well as CTC analyses can be performed as effective clinical practice tools in CRPC^31^. Although AR abnormalities are not limited to AR-V7, and there are various mechanisms of treatment resistance of AR target agents such as other splicing variants, AR amplification and mutation^32^, it is hoped that in the future all AR abnormalities could be monitored during CRPC treatment.

## Conclusion

This prospective study demonstrated that best BSI change at the end of the observation period while on ENZ treatment and PSA reduction at 3 months after ENZ administration were significant response biomarkers, and furthermore a negative CTC status at baseline was a predictive biomarker for ENZ efficacy in patients with mCRPC. The use of CTC analysis together with the routine monitoring of CTCs and AR abnormalities during treatment allow for a more personalized approach to the management of patients with mCRPC. The AdnaTest method would be useful in clinical practice as a POCT tool for evaluating AR-V7 status.

## Supplementary Information

Supplementary Information 1.

Supplementary Information 2.

## Data Availability

The datasets generated and analysed during the current study are available in the NBDC Human Database (National bioscience database center) repository, [https://humandbs.biosciencedbc.jp/en/ Research ID : hum0354].
