# Supplementary material for: Bone scan index (BSI) scoring by using bone scintigraphy and circulating tumor cells (CTCs): predictive factors for enzalutamide effectiveness in patients with castration-resistant prostate cancer and bone metastases
Source: Sci Rep. 2023 May 29;13:8704. doi: 10.1038/s41598-023-35790-5 (PMC10226993; doi:10.1038/s41598-023-35790-5)
Supplement: Supplementary file 1 — Supplementary Information 1. [file 41598_2023_35790_MOESM1_ESM.pptx]

## Slide 1
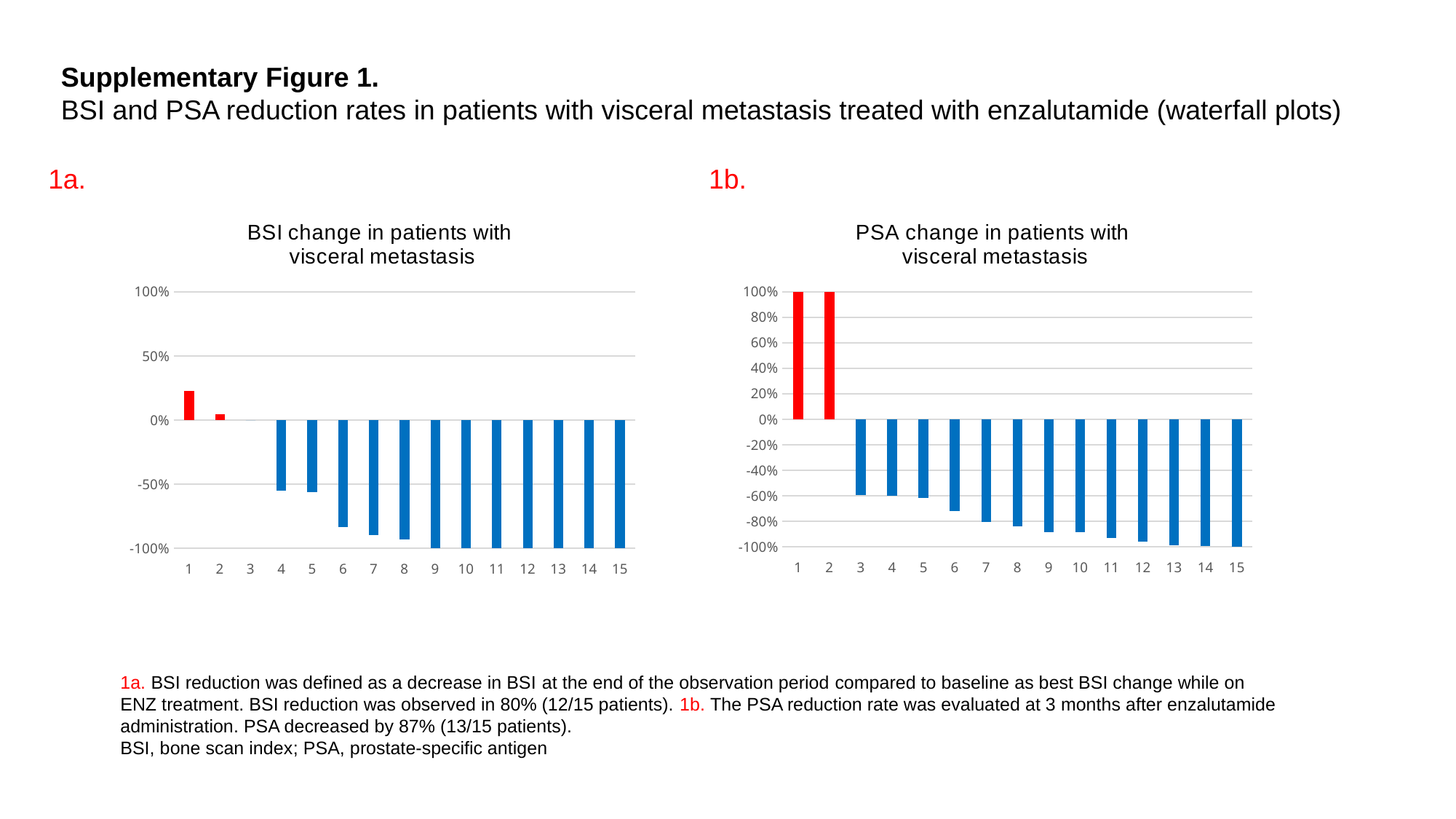

Supplementary Figure 1.
BSI and PSA reduction rates in patients with visceral metastasis treated with enzalutamide (waterfall plots)
1a.
1b.
### Chart: BSI change in patients with visceral metastasis
| Category | BSI change |
|---|---|
### Chart: PSA change in patients with visceral metastasis
| Category | PSA change |
|---|---|1a. BSI reduction was defined as a decrease in BSI at the end of the observation period compared to baseline as best BSI change while on ENZ treatment. BSI reduction was observed in 80% (12/15 patients). 1b. The PSA reduction rate was evaluated at 3 months after enzalutamide administration. PSA decreased by 87% (13/15 patients).
BSI, bone scan index; PSA, prostate-specific antigen

## Slide 2
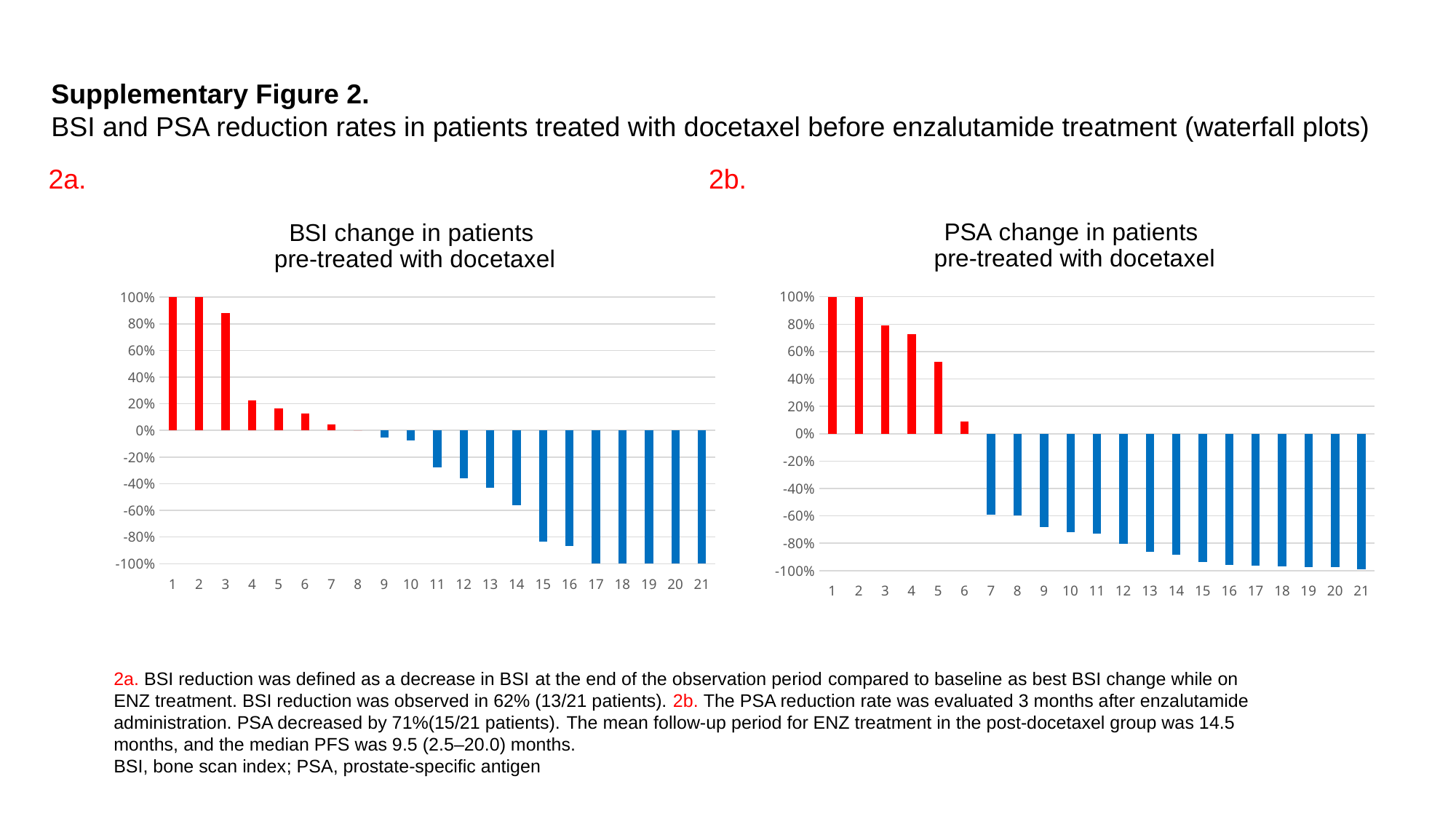

Supplementary Figure 2.
BSI and PSA reduction rates in patients treated with docetaxel before enzalutamide treatment (waterfall plots)
2a.
2b.
### Chart: PSA change in patients pre-treated with docetaxel
| Category | PSA change |
|---|---|
### Chart: BSI change in patients pre-treated with docetaxel
| Category | BSI change |
|---|---|2a. BSI reduction was defined as a decrease in BSI at the end of the observation period compared to baseline as best BSI change while on ENZ treatment. BSI reduction was observed in 62% (13/21 patients). 2b. The PSA reduction rate was evaluated 3 months after enzalutamide administration. PSA decreased by 71%(15/21 patients). The mean follow-up period for ENZ treatment in the post-docetaxel group was 14.5 months, and the median PFS was 9.5 (2.5–20.0) months.
BSI, bone scan index; PSA, prostate-specific antigen

## Slide 3
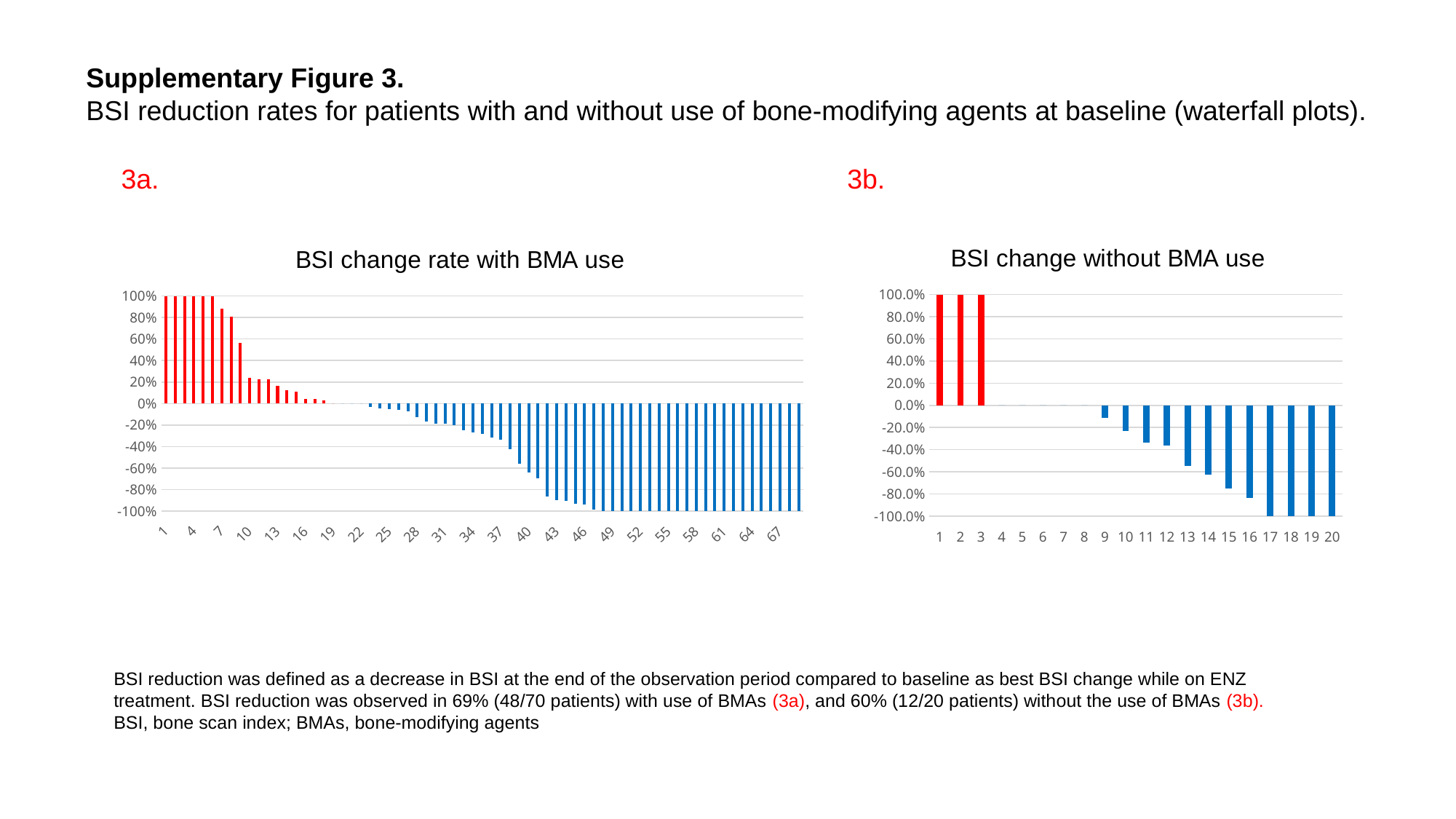

Supplementary Figure 3.
BSI reduction rates for patients with and without use of bone-modifying agents at baseline (waterfall plots).
3a.
3b.
### Chart: BSI change without BMA use
| Category | BSI change |
|---|---|
### Chart: BSI change rate with BMA use
| Category | |
|---|---|BSI reduction was defined as a decrease in BSI at the end of the observation period compared to baseline as best BSI change while on ENZ treatment. BSI reduction was observed in 69% (48/70 patients) with use of BMAs (3a), and 60% (12/20 patients) without the use of BMAs (3b).
BSI, bone scan index; BMAs, bone-modifying agents
