# Supplementary material for: Bone scan index (BSI) scoring by using bone scintigraphy and circulating tumor cells (CTCs): predictive factors for enzalutamide effectiveness in patients with castration-resistant prostate cancer and bone metastases
Source: Sci Rep. 2023 May 29;13:8704. doi: 10.1038/s41598-023-35790-5 (PMC10226993; doi:10.1038/s41598-023-35790-5)
Supplement: Supplementary file 2 — Supplementary Information 2. [file 41598_2023_35790_MOESM2_ESM.pptx]

## Slide 1
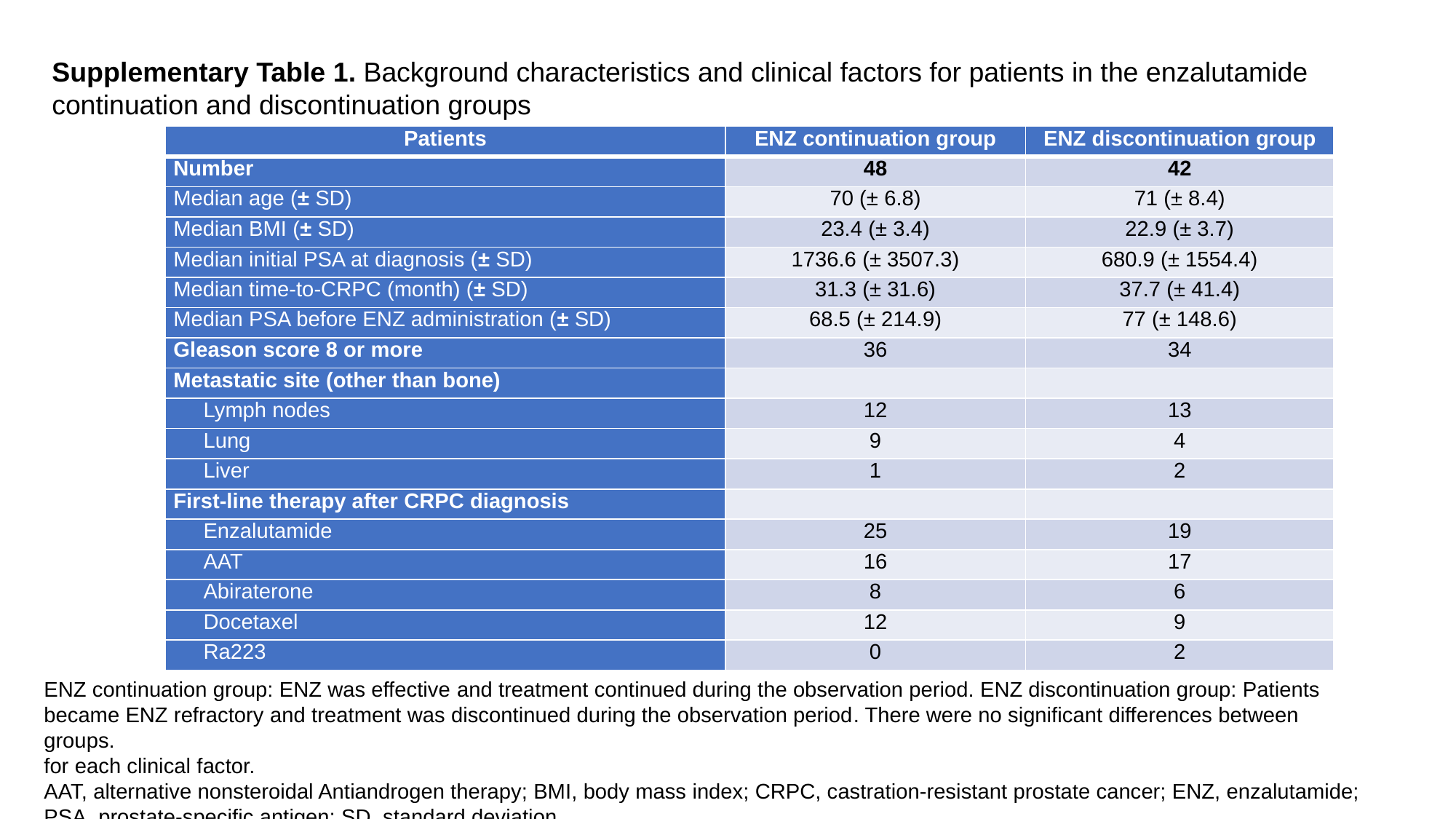

Supplementary Table 1. Background characteristics and clinical factors for patients in the enzalutamide
continuation and discontinuation groups
| Patients | ENZ continuation group | ENZ discontinuation group |
| --- | --- | --- |
| Number | 48 | 42 |
| Median age (± SD) | 70 (± 6.8) | 71 (± 8.4) |
| Median BMI (± SD) | 23.4 (± 3.4) | 22.9 (± 3.7) |
| Median initial PSA at diagnosis (± SD) | 1736.6 (± 3507.3) | 680.9 (± 1554.4) |
| Median time-to-CRPC (month) (± SD) | 31.3 (± 31.6) | 37.7 (± 41.4) |
| Median PSA before ENZ administration (± SD) | 68.5 (± 214.9) | 77 (± 148.6) |
| Gleason score 8 or more | 36 | 34 |
| Metastatic site (other than bone) | | |
| Lymph nodes | 12 | 13 |
| Lung | 9 | 4 |
| Liver | 1 | 2 |
| First-line therapy after CRPC diagnosis | | |
| Enzalutamide | 25 | 19 |
| AAT | 16 | 17 |
| Abiraterone | 8 | 6 |
| Docetaxel | 12 | 9 |
| Ra223 | 0 | 2 |
ENZ continuation group: ENZ was effective and treatment continued during the observation period. ENZ discontinuation group: Patients became ENZ refractory and treatment was discontinued during the observation period. There were no significant differences between groups.
for each clinical factor.
AAT, alternative nonsteroidal Antiandrogen therapy; BMI, body mass index; CRPC, castration-resistant prostate cancer; ENZ, enzalutamide; PSA, prostate-specific antigen; SD, standard deviation

## Slide 2
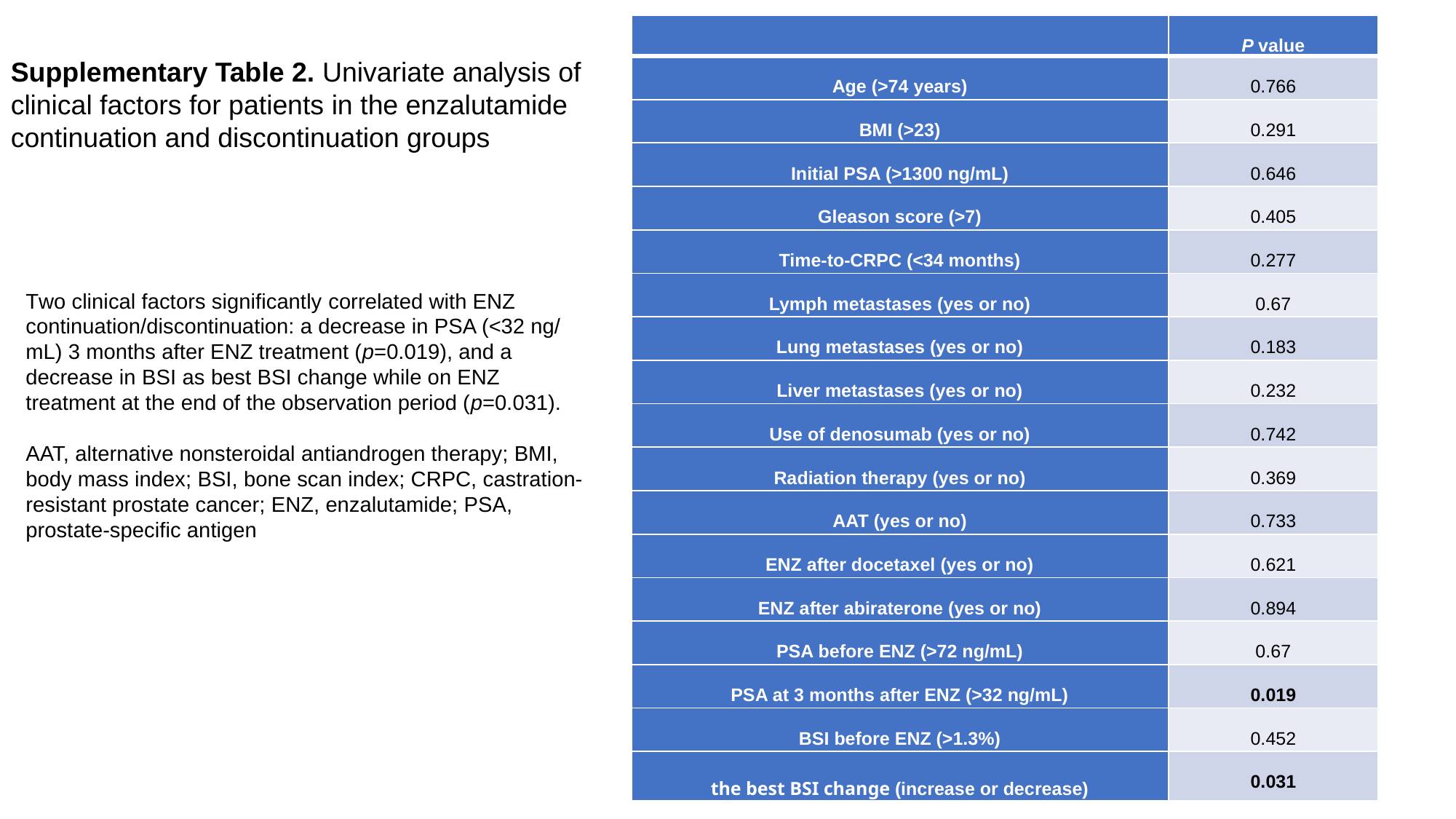

| | P value |
| --- | --- |
| Age (>74 years) | 0.766 |
| BMI (>23) | 0.291 |
| Initial PSA (>1300 ng/mL) | 0.646 |
| Gleason score (>7) | 0.405 |
| Time-to-CRPC (<34 months) | 0.277 |
| Lymph metastases (yes or no) | 0.67 |
| Lung metastases (yes or no) | 0.183 |
| Liver metastases (yes or no) | 0.232 |
| Use of denosumab (yes or no) | 0.742 |
| Radiation therapy (yes or no) | 0.369 |
| AAT (yes or no) | 0.733 |
| ENZ after docetaxel (yes or no) | 0.621 |
| ENZ after abiraterone (yes or no) | 0.894 |
| PSA before ENZ (>72 ng/mL) | 0.67 |
| PSA at 3 months after ENZ (>32 ng/mL) | 0.019 |
| BSI before ENZ (>1.3%) | 0.452 |
| the best BSI change (increase or decrease) | 0.031 |
Supplementary Table 2. Univariate analysis of clinical factors for patients in the enzalutamide continuation and discontinuation groups
Two clinical factors significantly correlated with ENZ continuation/discontinuation: a decrease in PSA (<32 ng/ mL) 3 months after ENZ treatment (p=0.019), and a decrease in BSI as best BSI change while on ENZ treatment at the end of the observation period (p=0.031).
AAT, alternative nonsteroidal antiandrogen therapy; BMI, body mass index; BSI, bone scan index; CRPC, castration-resistant prostate cancer; ENZ, enzalutamide; PSA, prostate-specific antigen

## Slide 3
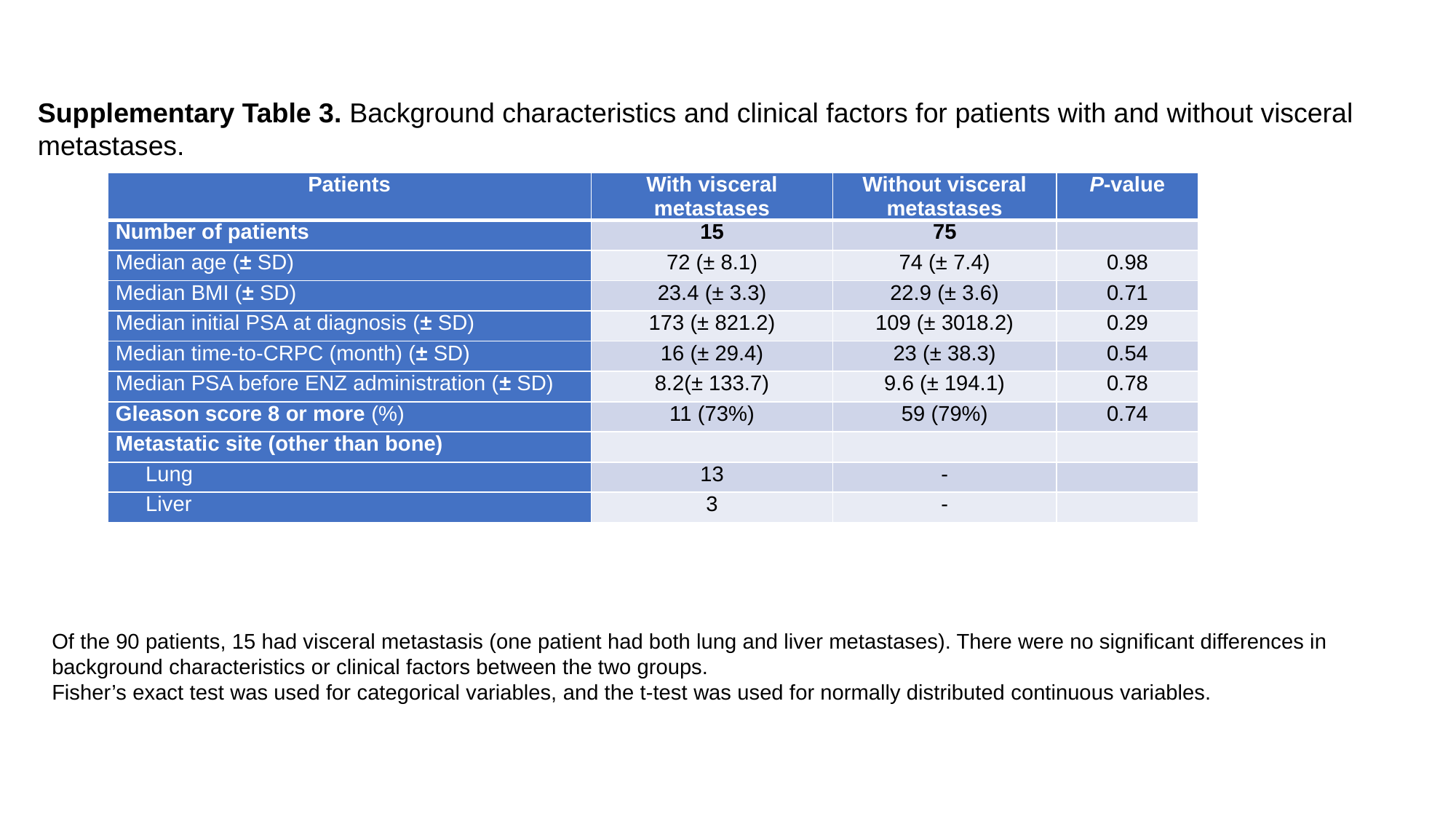

Supplementary Table 3. Background characteristics and clinical factors for patients with and without visceral
metastases.
| Patients | With visceral metastases | Without visceral metastases | P-value |
| --- | --- | --- | --- |
| Number of patients | 15 | 75 | |
| Median age (± SD) | 72 (± 8.1) | 74 (± 7.4) | 0.98 |
| Median BMI (± SD) | 23.4 (± 3.3) | 22.9 (± 3.6) | 0.71 |
| Median initial PSA at diagnosis (± SD) | 173 (± 821.2) | 109 (± 3018.2) | 0.29 |
| Median time-to-CRPC (month) (± SD) | 16 (± 29.4) | 23 (± 38.3) | 0.54 |
| Median PSA before ENZ administration (± SD) | 8.2(± 133.7) | 9.6 (± 194.1) | 0.78 |
| Gleason score 8 or more (%) | 11 (73%) | 59 (79%) | 0.74 |
| Metastatic site (other than bone) | | | |
| Lung | 13 | - | |
| Liver | 3 | - | |
Of the 90 patients, 15 had visceral metastasis (one patient had both lung and liver metastases). There were no significant differences in background characteristics or clinical factors between the two groups.
Fisher’s exact test was used for categorical variables, and the t-test was used for normally distributed continuous variables.

## Slide 4
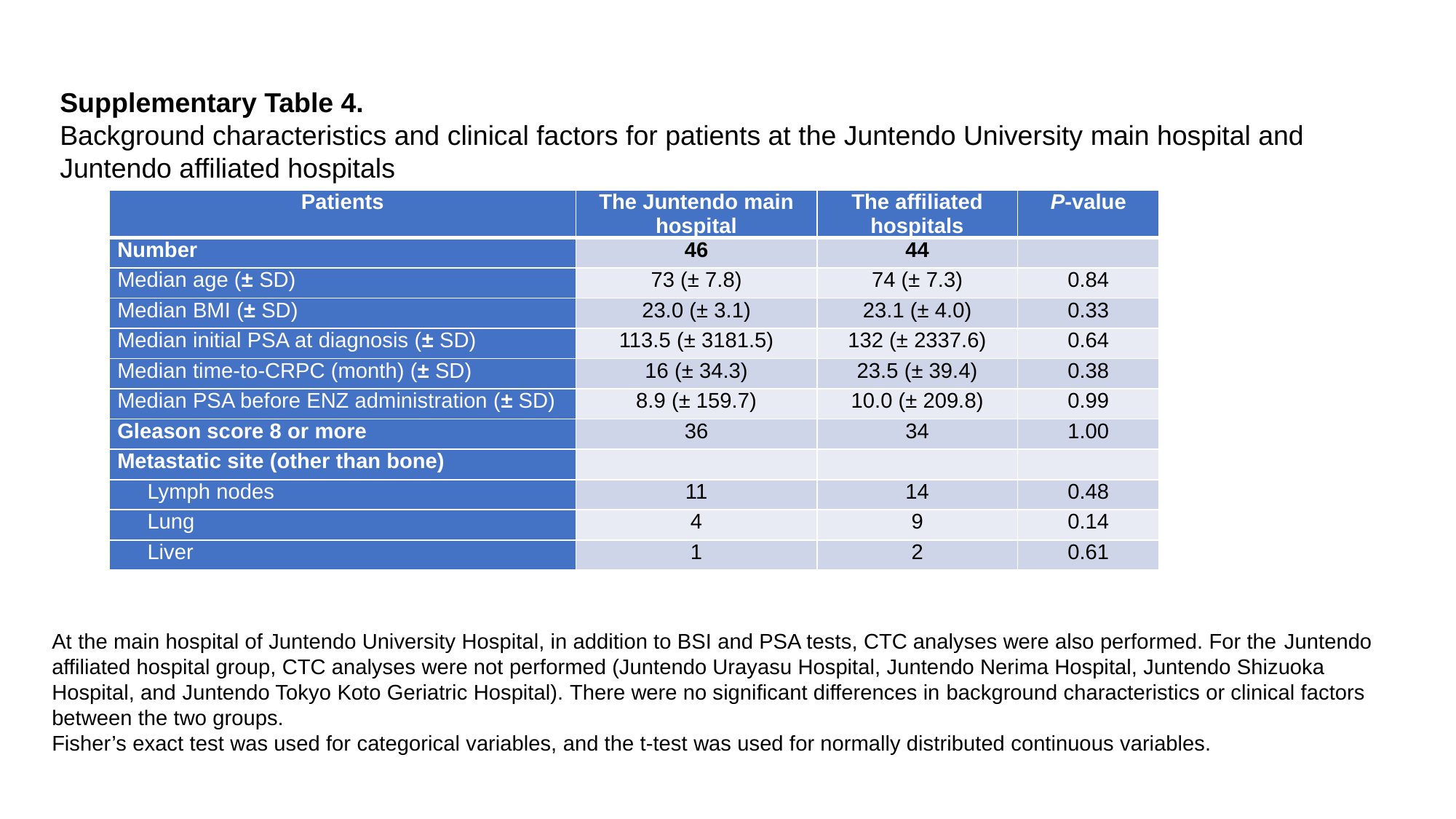

Supplementary Table 4.
Background characteristics and clinical factors for patients at the Juntendo University main hospital and
Juntendo affiliated hospitals
| Patients | The Juntendo main hospital | The affiliated hospitals | P-value |
| --- | --- | --- | --- |
| Number | 46 | 44 | |
| Median age (± SD) | 73 (± 7.8) | 74 (± 7.3) | 0.84 |
| Median BMI (± SD) | 23.0 (± 3.1) | 23.1 (± 4.0) | 0.33 |
| Median initial PSA at diagnosis (± SD) | 113.5 (± 3181.5) | 132 (± 2337.6) | 0.64 |
| Median time-to-CRPC (month) (± SD) | 16 (± 34.3) | 23.5 (± 39.4) | 0.38 |
| Median PSA before ENZ administration (± SD) | 8.9 (± 159.7) | 10.0 (± 209.8) | 0.99 |
| Gleason score 8 or more | 36 | 34 | 1.00 |
| Metastatic site (other than bone) | | | |
| Lymph nodes | 11 | 14 | 0.48 |
| Lung | 4 | 9 | 0.14 |
| Liver | 1 | 2 | 0.61 |
At the main hospital of Juntendo University Hospital, in addition to BSI and PSA tests, CTC analyses were also performed. For the Juntendo affiliated hospital group, CTC analyses were not performed (Juntendo Urayasu Hospital, Juntendo Nerima Hospital, Juntendo Shizuoka Hospital, and Juntendo Tokyo Koto Geriatric Hospital). There were no significant differences in background characteristics or clinical factors between the two groups.
Fisher’s exact test was used for categorical variables, and the t-test was used for normally distributed continuous variables.
